# Clinicopathological characteristics and stage-adjusted outcomes of sporadic pMMR/MSS early-onset colorectal cancer

**DOI:** 10.1186/s12876-026-04938-8

**Published:** 2026-05-21

**Authors:** Tomoyuki Momma, Hirokazu Okayama, Makoto Hasegawa, Takahiro Kawamata, Chiaki Takiguchi, Satoshi Fukai, Takahiro Sato, Misato Ito, Daisuke Ujiie, Shun Chida, Motonobu Saito, Koji Kono

**Affiliations:** 1 Department of Gastrointestinal Tract Surgery, Fukushima Medical University School of Medicine, 1 Hikarigaoka, Fukushima City, Fukushima 960-1295 Japan; 2 Department of Cancer Genome Medicine, Fukushima Medical University Hospital, 1 Hikarigaoka, Fukushima City, Fukushima 960-1295 Japan

**Keywords:** Colorectal cancer, Early-onset colorectal cancer (EOCRC), Late-onset colorectal cancer (LOCRC), Proficient mismatch repair/microsatellite stable (pMMR/MSS)

## Abstract

**Background:**

The global incidence of early-onset colorectal cancer (EOCRC) is increasing. The prognostic impact of EOCRC remains controversial, as it is largely confounded by hereditary syndromes and mismatch repair deficient (dMMR)/microsatellite instability-high (MSI-H) tumors. We aimed to evaluate the clinicopathological features and stage-adjusted outcomes of sporadic, proficient MMR/microsatellite stable (pMMR/MSS) EOCRC.

**Methods:**

We retrospectively reviewed 1,600 consecutive patients who underwent primary tumor resection for Stage 0–IV CRC. After excluding patients with known hereditary cancer syndromes or inflammatory bowel disease (IBD)-related CRC, as well as those with dMMR/MSI-H tumors or unknown MMR/MSI status, 1,218 patients with sporadic pMMR/MSS CRC were analyzed. Patients were stratified into EOCRC (< 50 years, *n* = 86) and late-onset CRC (LOCRC, ≥ 50 years, *n* = 1,132) cohorts. Cancer-specific survival (CSS) and time to recurrence (TTR) were evaluated.

**Results:**

Patients with EOCRC were more often diagnosed at an advanced stage than those with LOCRC (*P* < 0.001). Unadjusted CSS was worse in the EOCRC group (*P* = 0.042); however, multivariable analysis showed that EOCRC was not an independent predictor of worse CSS (adjusted hazard ratio [HR] 0.84, 95% confidence interval [CI] 0.49–1.37, *P* = 0.512). Among 987 patients with curatively resected Stage I–III disease, the EOCRC group received significantly more intensive perioperative treatments (*P* < 0.001). Unadjusted TTR did not significantly differ between the age groups (*P* = 0.510), and multivariable analysis showed a non-significant tendency toward a lower recurrence risk in patients with EOCRC (adjusted HR 0.52, 95% CI 0.23–1.03, *P* = 0.084).

**Conclusions:**

After exclusion of hereditary syndromes, IBD-related CRC, and dMMR/MSI-H tumors, pMMR/MSS EOCRC more often presented with advanced disease, but its stage-adjusted cancer-specific survival and recurrence outcomes did not significantly differ from those of LOCRC.

**Supplementary Information:**

The online version contains supplementary material available at 10.1186/s12876-026-04938-8.

## Introduction

Colorectal cancer (CRC) remains one of the most commonly diagnosed malignancies and a leading cause of cancer-related mortality worldwide [1]. Over the past few decades, the widespread implementation of screening programs, advances in multidisciplinary treatments, and improvements in lifestyle factors have led to a steady decline in both the incidence and mortality of CRC among individuals aged 50 years and older (late-onset CRC; LOCRC), particularly in many Western high-income countries [2, 3]. This favorable trend, however, is not uniform globally; in several Asian countries, including Japan, the incidence of LOCRC continues to rise, largely attributed to the cumulative effects of Westernized dietary habits and suboptimal screening adherence [4]. Concurrently, an important global trend has emerged: the incidence of early-onset CRC (EOCRC), defined as CRC diagnosed in individuals under 50 years of age, has been increasing steadily over the past few decades, not only in Western nations but also in Asian countries [4–7].

Accumulating evidence suggests that EOCRC exhibits a clinical and pathological profile distinct from that of LOCRC. EOCRC tumors are more frequently located in the distal colon and rectum, and they tend to present with aggressive histological features, such as poor differentiation, mucinous, or signet-ring cell histology [5, 8]. Furthermore, EOCRC is often diagnosed at more advanced stages, possibly due to a more aggressive tumor biology or delayed diagnosis. Importantly, the etiologic landscape of EOCRC is highly heterogeneous. Approximately 10 to 20% of EOCRC cases are associated with hereditary cancer susceptibility syndromes, most notably Lynch syndrome (LS), resulting in a significantly higher prevalence of DNA mismatch repair deficiency (dMMR) or high microsatellite instability (MSI-H) compared with LOCRC [5, 6]. These dMMR/MSI-H tumors are typically located in the right colon and frequently exhibit poorly differentiated or mucinous histology [9–11]. Although they are generally associated with a favorable stage-adjusted prognosis in the non-metastatic setting, they also have distinct therapeutic implications in metastatic disease because of their sensitivity to immune checkpoint inhibitors [12, 13]. dMMR/MSI-H and pMMR/MSS tumors exhibit fundamentally different genomic profiles, histological features, and survival outcomes, contributing to substantial heterogeneity within EOCRC that complicates survival analyses [14, 15]. Additionally, inflammatory bowel disease (IBD) accounts for another distinct subset of EOCRC [5, 15, 16]. Because of this heterogeneity, the reported survival outcomes for EOCRC remain highly inconsistent and controversial. Indeed, studies have variably reported worse, comparable, or even better prognoses relative to older patients [5, 8, 15]. This prognostic discrepancy is largely confounded by the admixture of hereditary syndromes, dMMR/MSI-H tumors, and IBD-related cases. Consequently, the outcomes of sporadic EOCRC, independent of major confounders, remain to be fully elucidated.

To address this critical knowledge gap and reduce major sources of prognostic confounding introduced by genetic predispositions and distinct tumor biology, we conducted a comprehensive analysis of a single-center cohort comprising over 1,200 patients who underwent primary tumor resection for Stage 0–IV CRC. We excluded individuals with hereditary cancer syndromes, including LS and familial adenomatous polyposis (FAP), as well as those with IBD-related and dMMR/MSI-H tumors. Thus, our study focuses on clinically sporadic, proficient mismatch repair/microsatellite stable (pMMR/MSS) CRC to compare the clinicopathological characteristics and outcomes between EOCRC and LOCRC, independent of major clinicopathological confounders. For prognostic evaluation, we assessed cancer-specific survival (CSS) in patients with Stage I–IV disease and specifically analyzed time to recurrence (TTR) in the subgroup of patients with Stage I–III CRC who underwent curative-intent resection. Crucially, we selected these cancer-focused endpoints rather than overall survival (OS) or relapse-free survival (RFS) to reduce confounding from age-related comorbidities and non-cancer mortality, which are more common in older populations [17, 18].

## Methods

### Study population

The source population comprised 1,600 consecutive patients who underwent primary tumor resection for histologically proven primary Stage 0–IV CRC at Fukushima Medical University Hospital between January 2004 and December 2025. To construct a clinically sporadic pMMR/MSS cohort and reduce major biological heterogeneity, we applied the following exclusion criteria: known hereditary cancer syndromes, IBD-related CRC, unknown MMR/MSI status, and dMMR/MSI-H tumors. Patients with hereditary cancer syndromes (*n* = 12) and those with IBD-related CRC (*n* = 12) were first excluded. To classify tumors as pMMR/MSS or dMMR/MSI-H for cohort definition, MMR/MSI status was assessed using immunohistochemistry (IHC) for MMR proteins (MLH1, MSH2, MSH6, and PMS2) and/or PCR-based MSI testing. Tumors showing loss of expression of at least one MMR protein were classified as dMMR, whereas tumors with intact expression of all four proteins were classified as pMMR. MSI testing results were obtained from the medical records; tumors reported as MSI-high or MSI-positive were classified as MSI-H, whereas those reported as MSI-low, MSI-negative, or microsatellite stable were classified as MSS. Routine MSI testing or MMR-IHC has been performed for virtually all patients undergoing tumor resection at our institution since late 2021; in earlier years, it was generally performed at the time of recurrence or when clinically indicated. Consequently, MMR/MSI information was available for 458 patients directly from their medical records. We additionally supplemented this with MMR-IHC results for 866 patients derived from our previous studies [11, 19–21]. In total, MMR/MSI status was evaluable for 1,324 tumors, while it remained unknown for 252 tumors. After excluding the 252 patients with unknown status and 106 patients identified as having dMMR/MSI-H tumors, a final cohort of 1,218 patients with Stage 0–IV sporadic, pMMR/MSS CRC was included. These patients were stratified by age at diagnosis into the EOCRC group (< 50 years, *n* = 86) and the LOCRC group (≥ 50 years, *n* = 1,132). Clinicopathological data were extracted from medical records. Tumor location was categorized into the right colon (from the cecum to the transverse colon), left colon (descending, sigmoid colon, and rectosigmoid junction), and rectum. Tumor staging was determined according to the Japanese Classification of Colorectal, Appendiceal, and Anal Carcinoma (JCCRC) [22]. For analytic consistency, baseline stage was defined as pathological stage in patients who underwent upfront surgery and as clinical stage in those who received preoperative therapy. In patients receiving preoperative therapy, clinical stage before treatment was used to approximate pretreatment disease extent, because postoperative pathological stage may be affected by treatment-related downstaging.

### Survival analysis

For survival analyses, cancer-specific survival (CSS) and time to recurrence (TTR) were evaluated. Patients who underwent surgery in 2025 were excluded from survival analyses to avoid including cases with insufficient follow-up duration for evaluating CSS or TTR. The CSS analysis included patients with Stage I–IV disease after exclusion of Stage 0 disease and patients who underwent surgery in 2025. The TTR analysis was restricted to curatively resected Stage I–III disease after exclusion of patients who underwent surgery in 2025. Curative (R0) resection was defined as the absence of macroscopic and microscopic residual tumor. CSS was defined as the time from surgery to CRC-related death or the last follow-up. TTR was defined as the time from the date of primary surgery to the date of first recurrence or the last follow-up. For CSS, deaths from causes other than CRC were treated as censored observations. For TTR, patients who died without documented recurrence were censored at the time of death. For all survival analyses, patients with Stage I and Stage II disease were combined into a single category (Stage I–II) because the limited number of outcome events in these early stages precluded stable separate estimates. For patients included in the TTR analysis, detailed information regarding neoadjuvant and adjuvant treatment regimens was collected. Neoadjuvant treatments were categorized as not received, short-course radiotherapy (SCRT), long-course chemoradiotherapy concurrent with capecitabine or S-1 (CRT), capecitabine and oxaliplatin or folinic acid, fluorouracil, and oxaliplatin (CAPOX/FOLFOX), and CAPOX/FOLFOX either before or after radiotherapy (CAPOX/FOLFOX + RT). Adjuvant treatments were categorized as not received, fluoropyrimidine monotherapy, and CAPOX/FOLFOX. The median follow-up period for the entire cohort was 49.8 months.

### Statistical analysis

Clinicopathological characteristics between the EOCRC and LOCRC groups were compared using Welch’s t-test for continuous variables. For categorical variables, Chi-square test or Fisher’s exact test was used, as appropriate, while the Chi-square test for trend was applied for ordinal variables. Survival curves for CSS and TTR were constructed using the Kaplan-Meier method, and differences between the groups were evaluated using the log-rank test. Univariable and multivariable Cox proportional hazards regression models were performed to identify independent prognostic factors, with results expressed as hazard ratios (HRs) and 95% confidence intervals (CIs). For Cox regression analyses performed in GraphPad Prism, *P*-values for individual coefficients were calculated using Wald statistics, whereas 95% CIs were derived using profile likelihood. Covariates for the multivariable Cox models were selected a priori based on clinical relevance and their potential roles as confounders, and the multivariable models included the following covariates: age group, sex, tumor location, histological type, tumor stage, R0 resection, and year of surgery (< 2010 vs. ≥2010) for the CSS analysis; and age group, sex, tumor location, histological type, tumor stage, neoadjuvant treatment, adjuvant treatment, and year of surgery for the TTR analysis. A two-sided *P*-value of < 0.05 was considered statistically significant. All statistical analyses were conducted using GraphPad Prism 9 (GraphPad Software, San Diego, CA).

## Results

### Study population and baseline patient characteristics

**Fig. 1:**
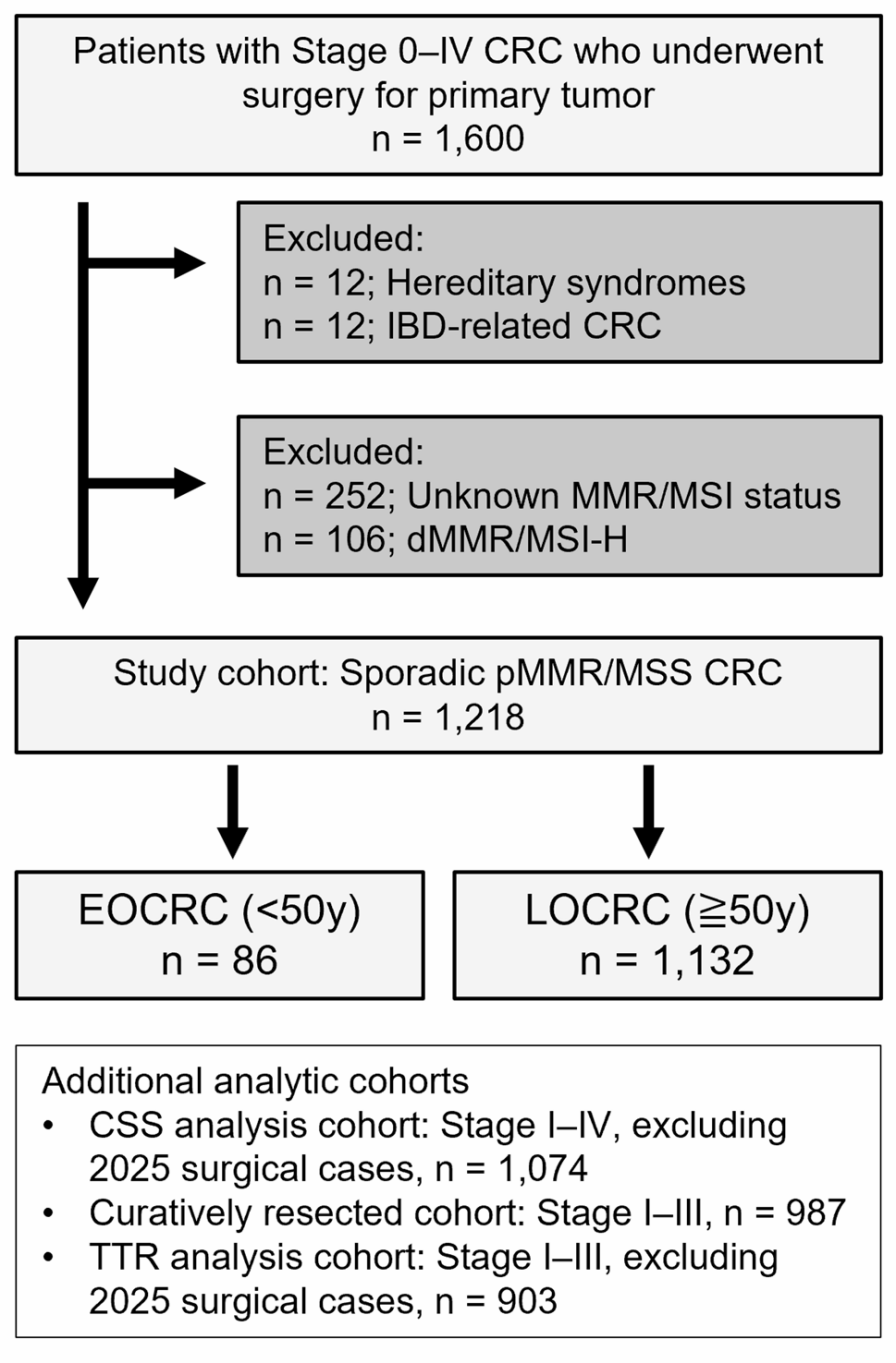
Flowchart of patient selection and analytic cohorts. Of 1,600 patients who underwent primary tumor resection for Stage 0–IV CRC, 12 patients with known hereditary cancer syndromes, 12 with IBD-related CRC, 252 with unknown MMR/MSI status, and 106 with dMMR/MSI-H tumors were excluded. The final study cohort comprised 1,218 patients with sporadic pMMR/MSS CRC, including 86 with EOCRC and 1,132 with LOCRC. Additional analytic cohorts were defined as follows: the CSS analysis cohort included patients with Stage I–IV disease after exclusion of 2025 surgical cases (*n* = 1,074); the curatively resected cohort included patients with Stage I–III disease who underwent R0 resection (*n* = 987); and the TTR analysis cohort included curatively resected Stage I–III patients after exclusion of 2025 surgical cases (*n* = 903)

As illustrated in Fig. 1, a total of 1,600 patients who underwent primary tumor resection for CRC were initially identified for this study. To establish a sporadic cohort and eliminate potential prognostic confounding factors, we sequentially excluded patients based on the following criteria: 12 patients with hereditary cancer syndromes (6 LS and 6 FAP), 12 patients with IBD-related CRC (11 with ulcerative colitis and 1 with Crohn disease), 252 patients with an unknown MMR/MSI status, and 106 patients with dMMR/MSI-H tumors. Following these exclusions, the final study cohort comprised 1,218 patients with sporadic, pMMR/MSS CRC. To further assess potential selection bias related to exclusion of the 252 patients with unknown MMR/MSI status, we compared their baseline clinicopathological characteristics with those of the included pMMR/MSS study cohort (Supplementary Table S1). No major difference in age distribution was observed between the two groups. However, the excluded cases were more frequently rectal, more often early stage, and more likely to have undergone R0 resection. Among the 1,218 patients in the study cohort, 86 (7.1%) were categorized into the EOCRC (< 50 years) group, and 1,132 (92.9%) into the LOCRC (≥ 50 years) group. Table 1 summarizes the baseline clinicopathological characteristics of the 1,218 patients. The mean age at diagnosis was 43.3 ± 5.1 years for the EOCRC group and 70.3 ± 9.3 years for the LOCRC group. The EOCRC cohort exhibited a significantly higher proportion of female patients compared to the LOCRC cohort (50.0% vs. 36.0%, *P* = 0.011). Notably, there were no significant differences between the age groups regarding primary tumor location (*P* = 0.124) or histological type (*P* = 0.128). However, patients with EOCRC presented with significantly more advanced disease (*P* < 0.001), including a higher prevalence of Stage III (36.0% vs. 32.4%) and Stage IV (29.1% vs. 13.3%) cancers. Consistent with this advanced stage at presentation, the rate of R0 resection was significantly lower in the EOCRC group than in the LOCRC group (77.9% vs. 88.3%, *P* = 0.005).

**Table 1:** Baseline clinicopathological characteristics of patients with pMMR/MSS colorectal cancer

	Total	EOCRC	LOCRC	*P*
	*n* = 1,218	*n* = 86 (7.1%)	*n* = 1,132 (92.9%)	
Age				**< 0.001**
Mean ± SD	68.4 ± 11.4	43.3 ± 5.1	70.3 ± 9.3	
Sex				**0.011**
Male	767 (63.0%)	43 (50.0%)	724 (64.0%)	
Female	451 (37.0%)	43 (50.0%)	408 (36.0%)	
Location				0.124
Right colon	374 (30.7%)	19 (22.1%)	355 (31.4%)	
Left colon	483 (39.7%)	40 (46.5%)	443 (39.1%)	
Rectum	361 (29.6%)	27 (31.4%)	334 (29.5%)	
Histological type				0.128
Well–Moderately differentiated	1137 (93.3%)	77 (89.5%)	1060 (93.6%)	
Poorly differentiated	19 (1.6%)	3 (3.5%)	16 (1.4%)	
Mucinous	59 (4.8%)	5 (5.8%)	54 (4.8%)	
Signet-ring cell	3 (0.2%)	1 (1.2%)	2 (0.2%)	
Baseline stage				**< 0.001**
Stage 0	49 (4.0%)	4 (4.7%)	45 (4.0%)	
Stage I	277 (22.7%)	11 (12.8%)	266 (23.5%)	
Stage II	319 (26.2%)	15 (17.4%)	304 (26.9%)	
Stage III	398 (32.7%)	31 (36.0%)	367 (32.4%)	
Stage IV	175 (14.4%)	25 (29.1%)	150 (13.3%)	
R0 resection				**0.005**
Yes	1067 (87.6%)	67 (77.9%)	1000 (88.3%)	
No	151 (12.4%)	19 (22.1%)	132 (11.7%)	

*pMMR/MSS* mismatch repair-proficient/microsatellite stable, *EOCRC *early-onset colorectal cancer, *LOCRC *late-onset colorectal cancer, *SD *standard deviation

### Cancer-specific survival in patients with stage I–IV disease

**Fig. 2:**
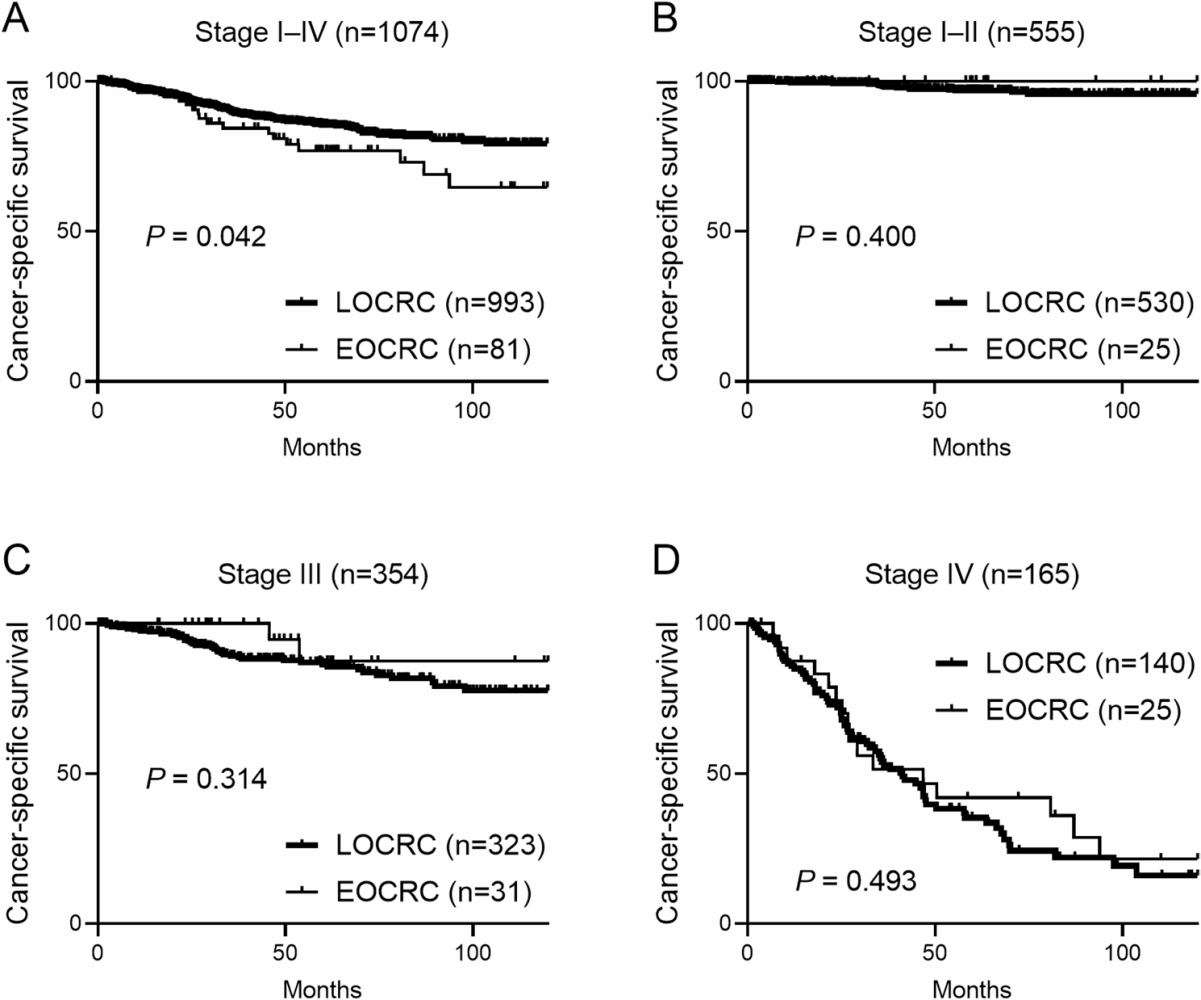
Kaplan-Meier estimates of cancer-specific survival. Survival curves comparing the EOCRC and LOCRC groups in the overall cohort (**A**), and stratified by tumor stage: Stage I–II (**B**), Stage III (**C**), and Stage IV (**D**). *P*-values were calculated using the log-rank test

As described in the Methods, 1,074 patients were eligible for the CSS analysis. During follow-up, 145 cancer-specific death events were observed, including 18 in the EOCRC group and 127 in the LOCRC group. Figure 2 demonstrates the Kaplan-Meier survival analyses for CSS across the study cohort. In the unstratified analysis of patients with stage I–IV disease (Fig. 2A), the EOCRC group (*n* = 81) exhibited significantly worse CSS compared to the LOCRC group (*n* = 993) (*P* = 0.042). However, to further account for the confounding by disease stage at presentation, we further performed stage-stratified survival analyses. Notably, the Kaplan-Meier curves for the two age groups demonstrated similar survival patterns within each individual stage category, including Stage I–II (Fig. 2B, *P* = 0.400), Stage III (Fig. 2C, *P* = 0.314), and Stage IV (Fig. 2D, *P* = 0.493), revealing no statistically significant differences in stage-specific CSS. These findings suggest that the inferior overall CSS observed in the unadjusted EOCRC cohort is primarily driven by the disproportionately high burden of advanced-stage disease at diagnosis.

To further investigate whether early-onset age serves as an independent prognostic factor for CSS, we performed univariable and multivariable Cox proportional hazards regression analyses (Table 2). In the univariable analysis, patients with EOCRC exhibited a higher risk of cancer-specific mortality compared to those with LOCRC (HR 1.66; 95% CI 0.98–2.64; *P* = 0.044). Other univariable factors associated with worse CSS included poor/mucinous/signet-ring cell histology, advanced tumor stages (Stage III and IV), and non-R0 resection. Crucially, after adjusting for these clinicopathological variables and the surgical era (≥ 2010 vs. <2010), early-onset age was no longer an independent predictor of worse CSS (adjusted HR 0.84; 95% CI 0.49–1.37; *P* = 0.512). In the multivariable model, only advanced disease stage and incomplete surgical resection remained as independent negative prognostic factors. These findings suggest that the worse unadjusted CSS observed in the EOCRC group was largely explained by a higher burden of advanced-stage disease at presentation.

**Table 2:** Univariable and multivariable cox proportional hazards models for cancer-specific survival in patients with stage I–IV pMMR/MSS colorectal cancer (*n* = 1,074)

	Univariable			Multivariable		
	HR	95% CI	*P*	HR	95% CI	*P*
Age group						
LOCRC	Reference			Reference		
EOCRC	1.66	0.98–2.64	**0.044**	0.84	0.49–1.37	0.512
Sex						
Male	Reference			Reference		
Female	0.93	0.66–1.29	0.660	1.04	0.74–1.46	0.824
Tumor location						
Right colon	Reference			Reference		
Left colon	1.15	0.78–1.72	0.498	0.82	0.55–1.25	0.348
Rectum	1.03	0.67–1.59	0.898	1.07	0.69–1.67	0.760
Histological type						
Wel/Mod	Reference			Reference		
Por/Muc/Sig	2.59	1.59–4.02	**< 0.001**	1.48	0.90–2.32	0.103
Baseline stage						
Stage I–II	Reference			Reference		
Stage III	5.50	3.08–10.43	**< 0.001**	5.41	3.02–10.29	**< 0.001**
Stage IV	37.87	22.24–69.64	**< 0.001**	21.24	9.53–46.83	**< 0.001**
Surgery						
R0	Reference			Reference		
Non-R0	14.24	10.21–19.91	**< 0.001**	2.19	1.21–4.28	**0.015**
Year of surgery						
≥ 2010	Reference			Reference		
< 2010	0.86	0.59–1.24	0.432	1.17	0.80–1.69	0.412

*P*-values for individual coefficients were calculated using Wald statistics, whereas 95% confidence intervals (CIs) were derived using profile likelihood

*pMMR/MSS* mismatch repair-proficient/microsatellite stable, *HR *hazard ratio, *EOCRC *early-onset colorectal cancer, *LOCRC *late-onset colorectal cancer, *Wel/Mod *well to moderately differentiated adenocarcinoma, *Por/Muc/Sig *poorly differentiated/mucinous adenocarcinoma or signet-ring cell carcinoma

### Clinicopathological features and treatment patterns in curatively resected stage I–III disease

To specifically evaluate the risk of disease recurrence, we restricted our subsequent analyses to a subgroup of 987 patients with Stage I–III CRC who underwent curative (R0) resection. Within this cohort, 57 patients (5.8%) were classified into the EOCRC group and 930 (94.2%) into the LOCRC group. The clinicopathological characteristics and detailed treatment patterns of this subgroup are summarized in Table 3. Consistent with the overall cohort, the EOCRC group in this localized disease setting had a significantly higher proportion of female patients compared to the LOCRC group (50.9% vs. 35.9%, *P* = 0.033) and presented with more advanced tumor stages, notably a higher prevalence of Stage III disease (54.4% vs. 38.9%, *P* = 0.026). Interestingly, while tumor location did not differ significantly in the overall Stage 0–IV cohort, within this curatively resected Stage I–III subgroup, EOCRC tumors were significantly more likely to be located in the left-sided colon or rectum, with a markedly lower frequency in the right-sided colon compared to LOCRC (15.9% vs. 31.3%, *P* = 0.046). Furthermore, real-world treatment strategies differed substantially between the two age groups. Patients with EOCRC were significantly more likely to receive intensive multimodal therapies. A higher proportion of the EOCRC group received neoadjuvant treatment (*P* < 0.001), with a particularly high utilization of oxaliplatin-containing regimens, including CAPOX/FOLFOX and CAPOX/FOLFOX + RT (15.8% in EOCRC vs. 5.4% in LOCRC). Similarly, the administration of adjuvant chemotherapy was significantly more frequent and intensive in the younger cohort (*P* < 0.001); nearly 60% of patients with EOCRC received some form of adjuvant therapy, with a strikingly higher proportion of CAPOX/FOLFOX regimens compared to older patients (31.6% vs. 6.8%).

**Table 3:** Clinicopathological characteristics of patients with curatively resected stage I–III pMMR/MSS colorectal cancer

	Total	EOCRC	LOCRC	*P*
	*n* = 987	*n* = 57 (5.8%)	*n* = 930 (94.2%)	
Age				**< 0.001**
Mean ± SD	69.1 ± 11.0	43.8 ± 5.1	70.6 ± 9.3	
Sex				**0.033**
Male	624 (63.2%)	28 (49.1%)	596 (64.1%)	
Female	363 (36.8%)	29 (50.9%)	334 (35.9%)	
Location				**0.046**
Right colon	300 (30.4%)	9 (15.9%)	291 (31.3%)	
Left colon	377 (38.2%)	27 (47.4%)	350 (37.6%)	
Rectum	310 (31.4%)	21 (36.8%)	289 (31.1%)	
Histological type				0.747
Well–Moderately differentiated	929 (94.1%)	52 (91.2%)	877 (94.3%)	
Poorly differentiated	10 (1.0%)	1 (1.8%)	9 (1.0%)	
Mucinous	46 (4.7%)	4 (7.2%)	42 (4.5%)	
Signet-ring cell	2 (0.2%)	0 (0.0%)	2 (0.2%)	
Baseline stage				**0.026**
Stage I	277 (28.1%)	11 (19.3%)	266 (28.6%)	
Stage II	317 (32.1%)	15 (26.3%)	302 (32.5%)	
Stage III	393 (39.8%)	31 (54.4%)	362 (38.9%)	
Neoadjuvant treatment				**< 0.001**
Not received	897 (90.9%)	46 (80.7%)	851 (91.5%)	
SCRT	14 (1.4%)	2 (3.5%)	12 (1.3%)	
CRT	16 (1.6%)	0 (0.0%)	16 (1.7%)	
CAPOX/FOLFOX	26 (2.6%)	7 (12.3%)	19 (2.0%)	
CAPOX/FOLFOX + RT	34 (3.4%)	2 (3.5%)	32 (3.4%)	
Adjuvant treatment				**< 0.001**
Not received	656 (66.5%)	23 (40.4%)	633 (68.1%)	
Fluoropyrimidine	250 (25.3%)	16 (28.1%)	234 (25.2%)	
CAPOX/FOLFOX	81 (8.2%)	18 (31.6%)	63 (6.8%)	

*pMMR/MSS *mismatch repair-proficient/microsatellite stable, *EOCRC *early-onset colorectal cancer, *LOCRC *late-onset colorectal cancer, *SD *standard deviation, *SCRT *short-course radiotherapy, *CRT *long-course chemoradiotherapy concurrent with capecitabine or S-1, *CAPOX/FOLFOX *capecitabine and oxaliplatin or folinic acid, fluorouracil, and oxaliplatin, *CAPOX/FOLFOX + RT *CAPOX/FOLFOX either before or after radiotherapy

### Time to recurrence and the impact of intensive treatments

**Fig. 3:**
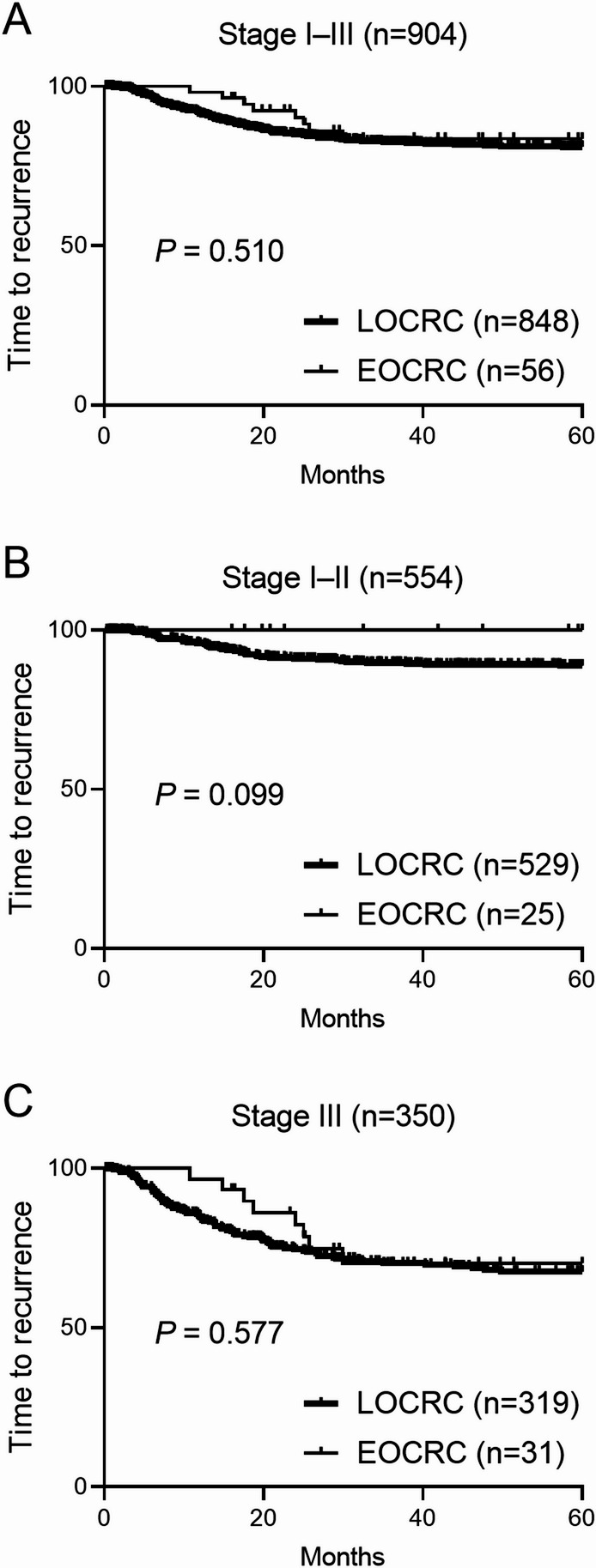
Kaplan-Meier estimates of time to recurrence. Survival curves comparing the EOCRC and LOCRC groups among patients who underwent curative resection for Stage I–III disease (**A**). Subgroup analyses stratified by tumor stage are shown for Stage I–II (**B**) and Stage III (**C**). *P*-values were calculated using the log-rank test

As described in the Methods, 903 patients were eligible for the TTR analysis. During follow-up, 146 recurrence events were observed, including 8 in the EOCRC group and 138 in the LOCRC group. Figure 3 depicts the Kaplan-Meier curves for TTR among patients who underwent curative resection for Stage I–III disease. In the unadjusted analysis of the overall Stage I–III cohort (Fig. 3A), TTR did not significantly differ between the EOCRC and LOCRC groups (*P* = 0.510). Stage-stratified analyses further confirmed no statistically significant differences in TTR between the two age groups in either Stage I–II (Fig. 3B, *P* = 0.099) or Stage III disease (Fig. 3C, *P* = 0.577). To rigorously assess the independent effect of early-onset age on recurrence risk while accounting for the profound disparities in real-world treatment intensity, we constructed univariable and multivariable Cox proportional hazards models (Table 4). In the univariable analysis, early-onset age was not significantly associated with TTR. However, in the multivariable model, after adjusting for tumor location, disease stage, the administration of various neoadjuvant and adjuvant treatments, and the surgical era, early-onset age demonstrated a trend toward a lower risk of recurrence, although this did not reach statistical significance (adjusted HR, 0.52; 95% CI, 0.23–1.03; *P* = 0.084). Furthermore, only rectal location and advanced disease stage (Stage III) remained as independent predictors of shorter TTR in the multivariable model (Table 4). These findings indicate that EOCRC was not independently associated with shorter TTR after adjustment for stage and recorded treatment variables. Whether differences in treatment intensity contributed to the observed TTR patterns cannot be determined from this retrospective analysis.

**Table 4:** Univariable and multivariable cox proportional hazards models for time to recurrence in patients with curatively resected stage I–III pMMR/MSS colorectal cancer (*n* = 903)

	Univariable			Multivariable		
	HR	95% CI	*P*	HR	95% CI	*P*
Age group						
LOCRC	Reference			Reference		
EOCRC	0.79	0.35–1.50	0.511	0.52	0.23–1.03	0.084
Sex						
Male	Reference			Reference		
Female	1.14	0.82–1.58	0.436	1.19	0.85–1.65	0.317
Tumor location						
Right colon	Reference			Reference		
Left colon	1.14	0.74–1.78	0.556	1.15	0.74–1.80	0.529
Rectum	1.85	1.23–2.84	**0.004**	1.65	1.05–2.61	**0.031**
Histological type						
Wel/Mod	Reference			Reference		
Por/Muc/Sig	1.65	0.89–2.80	0.086	1.36	0.71–2.41	0.323
Baseline stage						
Stage I–II	Reference			Reference		
Stage III	3.45	2.47–4.88	**< 0.001**	3.56	2.34–5.42	**< 0.001**
Neoadjuvant treatment						
Not received	Reference			Reference		
SCRT	1.80	0.44–4.75	0.316	1.05	0.25–3.07	0.933
CRT	2.78	1.09–5.78	**0.015**	1.14	0.41–2.67	0.773
CAPOX/FOLFOX	3.56	1.75–6.45	**< 0.001**	2.08	0.94–4.19	0.053
CAPOX/FOLFOX + RT	2.32	0.91–4.84	**0.044**	0.94	0.35–2.13	0.897
Adjuvant treatment						
Not received	Reference			Reference		
Fluoropyrimidine	1.52	1.06–2.17	**0.022**	0.85	0.56–1.28	0.429
CAPOX/FOLFOX	2.08	1.24–3.33	**0.004**	0.83	0.45–1.48	0.529
Year of surgery						
≥ 2010	Reference			Reference		
< 2010	0.96	0.66–1.37	0.830	1.14	0.76–1.68	0.503

*P*-values for individual coefficients were calculated using Wald statistics, whereas 95% confidence intervals (CIs) were derived using profile likelihood

*pMMR/MSS* mismatch repair-proficient/microsatellite stable, *HR *hazard ratio, *EOCRC *early-onset colorectal cancer, *LOCRC *late-onset colorectal cancer, *Wel/Mod *well to moderately differentiated adenocarcinoma, *Por/Muc/Sig *poorly differentiated/mucinous adenocarcinoma or signet-ring cell carcinoma, *SCRT *short-course radiotherapy, *CRT *long-course chemoradiotherapy concurrent with capecitabine or S-1, *CAPOX/FOLFOX *capecitabine and oxaliplatin or folinic acid, fluorouracil, and oxaliplatin, *CAPOX/FOLFOX + RT *CAPOX/FOLFOX either before or after radiotherapy

### Additional exploratory and sensitivity analyses

In an exploratory subgroup analysis within the EOCRC cohort using a younger age cutoff, no clear difference was observed between patients aged ≤ 40 years and those aged 41–49 years in CSS among Stage I–IV patients or in TTR among curatively resected Stage I–III patients (Supplementary Fig. S1A, B). We also performed a sensitivity analysis restricted to patients who underwent surgery through 2019 to evaluate patients with longer follow-up. This subset included 734 patients, with a median follow-up period of 62.3 months, and showed findings broadly consistent with the main CSS and TTR analyses (Supplementary Figs. S2A–D and S3A–C), although interpretation was limited by the reduced sample size and limited number of events in some subgroups.

## Discussion

In the present study, we examined the clinicopathological features and outcomes of EOCRC after reducing major sources of clinical and biological heterogeneity that have complicated prior epidemiological studies. By excluding patients with known hereditary syndromes (LS and FAP), IBD-related CRC, and dMMR/MSI-H tumors, we constructed a clinically sporadic pMMR/MSS cohort. Using CSS and TTR as cancer-specific endpoints, which may reduce confounding from age-related comorbidities and non-cancer mortality [15, 17, 18], we found that EOCRC was more often diagnosed at an advanced stage, whereas early-onset age itself was not independently associated with worse CSS or shorter TTR after adjustment for clinicopathological factors.

Consistent with numerous previous epidemiological reports, our baseline clinicopathological analyses revealed that patients with EOCRC were significantly more likely to present with advanced-stage disease [5, 6]. Furthermore, we observed a higher frequency of female patients in the EOCRC group, a demographic trend that has been similarly highlighted in several recent studies [3, 23]. The more advanced stage at presentation in EOCRC is likely to be multifactorial. Because younger individuals have historically been outside the target population for routine colorectal cancer screening, opportunities to detect asymptomatic or early-stage tumors may be reduced [7]. Diagnostic delay may also contribute. In younger adults, symptoms such as rectal bleeding, changes in bowel habits, or abdominal pain may initially be attributed to benign conditions by patients or healthcare providers, potentially delaying diagnosis [24, 25]. Although our dataset did not include detailed information on screening history, presenting symptoms, or the interval from symptom onset to diagnosis, these factors may partly explain the more advanced stage at presentation observed in the EOCRC group. Although several studies have reported that EOCRC typically exhibits aggressive histopathological features and left-sided predominance, our data demonstrated no significant differences in histological type and tumor location in the overall cohort, although right-sided tumors were less frequent in the localized EOCRC setting. The left-sided predominance observed only in the localized EOCRC subgroup should be interpreted as a subgroup-specific and hypothesis-generating finding. One possible explanation is that left-sided and rectal tumors are more likely to cause overt symptoms, which may prompt diagnostic evaluation before metastatic dissemination becomes apparent. This partial clinicopathological similarity between age groups may be partly attributable to the strict exclusion of dMMR/MSI-H and IBD-related tumors, both of which are known to frequently exhibit mucinous, poorly differentiated or signet-ring cell histological types [15, 16]. This observation is consistent with findings of Cercek et al., who demonstrated that when analyses were exclusively limited to MSS tumors, the histopathological characteristics between EOCRC and LOCRC became indistinguishable, indicating that the previously reported aggressive histology in young patients was largely confounded by the higher prevalence of dMMR/MSI-H tumors [26]. However, relatively few studies have specifically evaluated cancer-specific outcomes in surgically treated cohorts restricted to sporadic pMMR/MSS CRC. The present study extends these prior observations by assessing CSS and TTR using stage-stratified and multivariable analyses in a consecutive surgical cohort.

Our stage-stratified survival analyses and multivariable Cox regression models indicated that the initially observed inferior CSS was primarily driven by the advanced stage at presentation and subsequent incomplete surgical resection in the EOCRC group. The lower R0 resection rate in the EOCRC group likely reflects the higher proportion of Stage IV disease, in which R0 resection requires complete removal of both the primary tumor and metastatic lesions. Therefore, unresectable metastatic disease directly limits the likelihood of complete resection. Consistent with this clinical context, non-R0 resection remained independently associated with worse CSS in the multivariable model. Furthermore, to reduce confounding related to metastatic presentation, we evaluated TTR exclusively in patients who underwent curative-intent resection for localized disease. The EOCRC group was not associated with an increased risk of recurrence, although the multivariable model showed a non-significant tendency toward a numerically lower recurrence risk. These observations suggest that early-onset disease is not independently associated with a poorer prognosis after accounting for stage and other clinicopathological factors, which is consistent with several recent studies. For instance, a systematic review and meta-analysis reported no clear survival disadvantage for EOCRC despite its association with more advanced-stage disease and MSI-H tumors [27]. Similarly, analyses of the US National Cancer Database and a nationwide Swedish cohort showed that younger patients did not have inferior stage-adjusted survival, and in some settings had more favorable adjusted outcomes [28, 29]. The importance of molecular confounding was further highlighted in the ACCENT database analysis by Jin et al., in which the apparent prognostic advantage of early-onset age in Stage III disease was attenuated after adjusting for key molecular markers, such as MMR and BRAF status [30]. Together with prior MSS-restricted observations [26], these studies support the interpretation that age alone is unlikely to fully explain outcome differences between EOCRC and LOCRC. Although these studies differed in patient selection, study design, and endpoints, our findings are broadly consistent with their overall direction. By restricting our cohort to sporadic pMMR/MSS tumors, our study extended these observations to a real-world single-center cohort, suggesting that the oncological outcomes of EOCRC are similar to those of LOCRC when major genomic confounders are reduced. Additional exploratory and sensitivity analyses using a younger age cutoff and a longer-follow-up subset showed findings broadly consistent with the main analyses; however, these analyses should be regarded as supportive rather than confirmatory, given the small number of EOCRC patients and limited number of events in some subgroups.

The non-significant tendency toward a lower recurrence risk in the EOCRC group observed in our multivariable model requires careful interpretation. Consistent with previous studies [31–34], younger patients in our cohort were significantly more likely to receive intensive therapies, including oxaliplatin-containing regimens such as CAPOX and FOLFOX. Although our model was adjusted for the type of neoadjuvant and adjuvant regimens, as well as year of surgery, the present dataset did not include detailed information on treatment adherence, relative dose intensity, treatment completion, treatment modification, or long-term toxicity. Therefore, residual confounding by treatment intensity cannot be excluded. Historically, because EOCRC has been considered to exhibit aggressive tumor biology and worse outcomes, clinicians have often employed empirical treatment escalation [8, 15]. Furthermore, large-scale clinical trials, including the IDEA pooled analysis, suggest that younger patients tend to achieve higher treatment completion rates and dose intensities than older patients, possibly because of fewer age-related comorbidities and better general health [18]. In contrast to the findings from the ACCENT database [30], a post-hoc subgroup analysis of the IDEA trials demonstrated that despite better treatment adherence, early-onset age was associated with a significantly higher relapse rate [18]. However, this adverse prognostic association was limited to the high-risk Stage III subgroup, with no clear age-related survival difference in patients with Stage II or low-risk Stage III disease. Furthermore, it should be noted that their cohort included dMMR/MSI-H tumors, which are biologically distinct from the pMMR/MSS population examined in the present study. Taken together, these considerations suggest that the observed TTR pattern in our cohort should not be interpreted as evidence of more favorable tumor biology in EOCRC. The numerically lower recurrence risk should instead be interpreted cautiously, as it may reflect differences in treatment exposure or residual confounding. Large-scale population-based studies have also shown that young adults with CRC often receive more intensive systemic treatments, with limited or unmatched survival benefit compared to older patients [5, 31–33]. Indeed, international consensus guidelines recommend that oncologic treatment should not differ based solely on early-onset age [24]. Future real-world studies should incorporate detailed metrics of cumulative dose, treatment modifications, and long-term toxicities to clarify whether such intensive therapies provide meaningful survival benefit for young patients or expose them to unnecessary harm.

We acknowledge several limitations in the present study. First, the retrospective, single-center design inherently introduces selection bias. Because this study was conducted at a Japanese tertiary referral center, potential referral bias for complex or advanced cases may limit the generalizability of our findings to other ethnic populations and healthcare settings. External validation in multi-institutional or population-based cohorts will be needed. Second, several aspects of cohort definition and staging may have introduced additional heterogeneity or selection bias. Baseline stage was defined using pathological stage in patients who underwent upfront surgery and clinical stage in those who received preoperative treatment. Although this approach was intended to approximate pretreatment disease extent and avoid excluding patients treated with preoperative therapy, clinical staging is less precise than pathological staging and may have introduced some misclassification, particularly because patients with EOCRC were more likely to receive preoperative treatment. In addition, exclusion of 252 patients with unknown MMR/MSI status may have introduced selection bias. Comparison of these excluded cases with the included pMMR/MSS study cohort showed no major difference in age distribution; however, the excluded group was enriched for rectal tumors, early-stage disease, and R0 resections, suggesting that missing MMR/MSI status was not entirely random. One possible explanation is that MMR/MSI testing may have been performed more consistently in patients with surgically resected Stage II or more advanced CRC, whereas in Stage 0–I disease, where MMR/MSI status is less likely to affect immediate postoperative management, testing may have been omitted in some cases. Accordingly, exclusion of these cases could have introduced selection bias, particularly with respect to tumor location and stage distribution. Therefore, this selection pattern should be considered when interpreting and generalizing our findings, particularly for patients with rectal tumors, early-stage disease, or R0 resection. Third, the long study period and limited number of events should be considered. Temporal changes in diagnostic practice, perioperative treatment strategies, and supportive care may have introduced residual confounding that could not be fully captured by adjustment for year of surgery and recorded treatment categories. In addition, the relatively small number of EOCRC patients and outcome events, particularly the limited number of recurrence events in the EOCRC subgroup in the TTR analysis, may have limited the precision, stability, and robustness of the multivariable estimates, warranting cautious interpretation. Fourth, although dMMR/MSI-H tumors were excluded, comprehensive molecular profiling was not available for the entire cohort. In particular, RAS and BRAF mutation data were unavailable for a substantial proportion of cases because routine testing was introduced only in the later years of the study period. Moreover, only a very small number of BRAF-mutant tumors remained in the final pMMR/MSS cohort, limiting the feasibility of meaningful adjustment for these markers without substantial case loss and potential temporal bias. Similarly, the TTR analysis could not fully account for treatment intensity, because detailed data on treatment adherence, relative dose intensity, treatment completion, treatment modification, and long-term toxicity were unavailable. Finally, several unmeasured factors could not be evaluated. Although we excluded well-defined hereditary syndromes, we lacked detailed data on environmental and lifestyle factors, such as obesity and dietary habits, as well as potential familial clustering that does not meet clinical syndromic criteria. In addition, the lack of detailed information regarding screening history, presenting symptoms, and the interval from symptom onset to diagnosis limits our ability to fully explore the underlying factors contributing to the advanced stage at presentation frequently observed in the EOCRC group.

## Conclusions

In this single-center retrospective cohort, after exclusion of hereditary syndromes, IBD, and dMMR/MSI-H tumors, EOCRC in the pMMR/MSS setting was more often diagnosed at an advanced stage, but early-onset age was not independently associated with worse CSS or shorter TTR. These findings suggest that the less favorable unadjusted outcomes observed in EOCRC are largely explained by stage at presentation rather than age itself.

## Supplementary Information

Supplementary Material 1.

## Data Availability

The data presented in this study are available on reasonable request from the corresponding author.

## Abbreviations

CRC: Colorectal cancer
EOCRC: Early-onset colorectal cancer
LOCRC: Late-onset colorectal cancer
MMR: DNA Mismatch repair
dMMR: MMR deficient
MSI-H: Microsatellite instability-high
pMMR: MMR proficient
MSS: Microsatellite stable
IBD: Inflammatory bowel disease
CSS: Cancer-specific survival
TTR: Time to recurrence
HR: Hazard ratio
CI: Confidence interval
LS: Lynch syndrome
FAP: Familial adenomatous polyposis
OS: Overall survival
RFS: Relapse-free survival
IHC: Immunohistochemistry
JCCRC: Japanese Classification of Colorectal, Appendiceal, and Anal Carcinoma
SCRT: Short-course radiotherapy
CRT: Chemoradiotherapy
CAPOX: Capecitabine and oxaliplatin
FOLFOX: Folinic acid, fluorouracil, and oxaliplatin
RT: Radiotherapy

## References

[1] Bray F, Laversanne M, Sung H, Ferlay J, Siegel RL, Soerjomataram I, Jemal A. Global cancer statistics 2022: GLOBOCAN estimates of incidence and mortality worldwide for 36 cancers in 185 countries. CA Cancer J Clin. 2024;74(3):229–63. 38572751 10.3322/caac.21834

[2] Dekker E, Tanis PJ, Vleugels JLA, Kasi PM, Wallace MB. Colorectal cancer. Lancet. 2019;394(10207):1467–80. 31631858 10.1016/S0140-6736(19)32319-0

[3] Siegel RL, Wagle NS, Star J, Kratzer TB, Smith RA, Jemal A. Colorectal cancer statistics, 2026. CA Cancer J Clin. 2026;76(2):e70067. 41769777 10.3322/caac.70067 PMC12951547

[4] Sung H, Siegel RL, Laversanne M, Jiang C, Morgan E, Zahwe M, Cao Y, Bray F, Jemal A. Colorectal cancer incidence trends in younger versus older adults: an analysis of population-based cancer registry data. Lancet Oncol. 2025;26(1):51–63. 39674189 10.1016/S1470-2045(24)00600-4 PMC11695264

[5] Akimoto N, Ugai T, Zhong R, Hamada T, Fujiyoshi K, Giannakis M, Wu K, Cao Y, Ng K, Ogino S. Rising incidence of early-onset colorectal cancer - a call to action. Nat Rev Clin Oncol. 2021;18(4):230–43. 33219329 10.1038/s41571-020-00445-1 PMC7994182

[6] Sinicrope FA. Increasing Incidence of Early-Onset Colorectal Cancer. N Engl J Med. 2022;386(16):1547–58. 35443109 10.1056/NEJMra2200869

[7] Patel SG, Karlitz JJ, Yen T, Lieu CH, Boland CR. The rising tide of early-onset colorectal cancer: a comprehensive review of epidemiology, clinical features, biology, risk factors, prevention, and early detection. Lancet Gastroenterol Hepatol. 2022;7(3):262–74. 35090605 10.1016/S2468-1253(21)00426-X

[8] Char SK, O’Connor CA, Ng K. Early-onset gastrointestinal cancers: comprehensive review and future directions. Br J Surg. 2025;112(7). 10.1093/bjs/znaf102. 10.1093/bjs/znaf102 PMC13234757 40624747

[9] Turk A, Mondaca S, Nervi B, Morris AD, Finer Z, Holowatyj AN. Early-Onset Colorectal Cancer: From Genetic Discovery to Clinical Innovation. Am Soc Clin Oncol Educ Book. 2025;45(3):e473618. 40638868 10.1200/EDBK-25-473618 PMC13131272

[10] Jin Z, Sinicrope FA. Prognostic and Predictive Values of Mismatch Repair Deficiency in Non-Metastatic Colorectal Cancer. Cancers (Basel). 2021;13(2):300. 10.3390/cancers13020300 PMC7830023 33467526

[11] Momma T, Okayama H, Hayashishita S, Suzuki H, Katagata M, Matsumoto T, Ujiie D, Chida S, Sakamoto W, Kono K. Prognostic Impact of Histologic Subtypes in Mismatch Repair-Deficient/Microsatellite Instability-High Colorectal Cancer: A Single-Center Retrospective Study of 1127 Stage 0–IV Patients. Ann Gastroenterol Surg. 2025;10(3):760–76. 10.1002/ags3.70159. 10.1002/ags3.70159 PMC13178275 42146830

[12] Jin Z, Sinicrope FA. Mismatch Repair-Deficient Colorectal Cancer: Building on Checkpoint Blockade. J Clin Oncol. 2022;40(24):2735–50. 35649217 10.1200/JCO.21.02691 PMC9390830

[13] Sveen A, Kopetz S, Lothe RA. Biomarker-guided therapy for colorectal cancer: strength in complexity. Nat Rev Clin Oncol. 2020;17(1):11–32. 31289352 10.1038/s41571-019-0241-1 PMC7577509

[14] Tang J, Peng W, Tian C, Zhang Y, Ji D, Wang L, Jin K, Wang F, Shao Y, Wang X, et al. Molecular characteristics of early-onset compared with late-onset colorectal cancer: a case controlled study. Int J Surg. 2024;110(8):4559–70. 38742845 10.1097/JS9.0000000000001584 PMC11326018

[15] Gandini A, Taieb J, Blons H, Netter J, Laurent-Puig P, Gallois C. Early-Onset colorectal Cancer: From the laboratory to the clinic. Cancer Treat Rev. 2024;130:102821. 39236404 10.1016/j.ctrv.2024.102821

[16] Arif AA, Chahal D, Ladua GK, Bhang E, Salh B, Rosenfeld G, Loree JM, Donnellan F. Hereditary and Inflammatory Bowel Disease-Related Early Onset Colorectal Cancer Have Unique Characteristics and Clinical Course Compared with Sporadic Disease. Cancer Epidemiol Biomarkers Prev. 2021;30(10):1785–91. 34301727 10.1158/1055-9965.EPI-21-0507

[17] Kanter K, Fish M, Mauri G, Horick NK, Allen JN, Blaszkowsky LS, Clark JW, Ryan DP, Nipp RD, Giantonio BJ, et al. Care Patterns and Overall Survival in Patients With Early-Onset Metastatic Colorectal Cancer. JCO Oncol Pract. 2021;17(12):e1846–55. 34043449 10.1200/OP.20.01010

[18] Fontana E, Meyers J, Sobrero A, Iveson T, Shields AF, Taieb J, Yoshino T, Souglakos I, Smyth EC, Lordick F, et al. Early-Onset Colorectal Adenocarcinoma in the IDEA Database: Treatment Adherence, Toxicities, and Outcomes With 3 and 6 Months of Adjuvant Fluoropyrimidine and Oxaliplatin. J Clin Oncol. 2021;39(36):4009–19. 34752136 10.1200/JCO.21.02008 PMC8677996

[19] Suzuki H, Okayama H, Nakajima S, Saito K, Kanoda R, Maruyama Y, Matsuishi A, Matsumoto T, Ito M, Chida S et al. GALNT7 stratifies dMMR/MSI colorectal cancer into distinct molecular subsets associated with prognosis and PD-L1 expression. Cancer Res Commun. 2025;5(9):1530–40. 10.1158/2767-9764.CRC-25-0270 PMC12412012 40824763

[20] Matsumoto T, Okayama H, Nakajima S, Saito K, Ito M, Kaneta A, Kanke Y, Onozawa H, Hayase S, Fujita S, et al. SH2D4A downregulation due to loss of chromosome 8p is associated with poor prognosis and low T cell infiltration in colorectal cancer. Br J Cancer. 2022;126(6):917–26. 34893760 10.1038/s41416-021-01660-y PMC8927606

[21] Noda M, Okayama H, Tachibana K, Sakamoto W, Saito K, Thar Min AK, Ashizawa M, Nakajima T, Aoto K, Momma T, et al. Glycosyltransferase Gene Expression Identifies a Poor Prognostic Colorectal Cancer Subtype Associated with Mismatch Repair Deficiency and Incomplete Glycan Synthesis. Clin Cancer Res. 2018;24(18):4468–81. 29844132 10.1158/1078-0432.CCR-17-3533

[22] Japanese Society for Cancer of the C, Rectum. Japanese Classification of Colorectal, Appendiceal, and Anal Carcinoma: the 3d English Edition [Secondary Publication]. J Anus Rectum Colon. 2019;3(4):175–95. 31768468 10.23922/jarc.2019-018 PMC6845287

[23] Akkus E, Karaoglan BB, Kayaalp M, Turmus U, Akyol C, Utkan G. Stage-specific characterization of early-onset colorectal cancer: Localized and synchronous metastatic disease. Int J Cancer. 2025;156(12):2340–51. 39887374 10.1002/ijc.35336 PMC12008821

[24] Cavestro GM, Mannucci A, Balaguer F, Hampel H, Kupfer SS, Repici A, Sartore-Bianchi A, Seppala TT, Valentini V, Boland CR, et al. Delphi Initiative for Early-Onset Colorectal Cancer (DIRECt) International Management Guidelines. Clin Gastroenterol Hepatol. 2023;21(3):581–603. e533. 36549470 10.1016/j.cgh.2022.12.006 PMC11207185

[25] Liao CK, Hsu YJ, Chern YJ, Yu YL, Lin YC, Hsieh PS, Chiang JM, You JF. Differences in characteristics and outcomes between early-onset colorectal cancer and late-onset colorectal cancers. Eur J Surg Oncol. 2024;50(12):108687. 39288563 10.1016/j.ejso.2024.108687

[26] Cercek A, Chatila WK, Yaeger R, Walch H, Fernandes GDS, Krishnan A, Palmaira L, Maio A, Kemel Y, Srinivasan P, et al. A Comprehensive Comparison of Early-Onset and Average-Onset Colorectal Cancers. J Natl Cancer Inst. 2021;113(12):1683–92. 34405229 10.1093/jnci/djab124 PMC8634406

[27] Huang QS, Yu XZ, Zhao R, Huang LB, Wen J, Yang L. Clinicopathological characteristics and biomarker alterations in early-onset vs. late-onset colorectal cancer: a systematic review and meta-analysis. Int J Surg. 2026;112(1):1840–54. 40956186 10.1097/JS9.0000000000003463 PMC12825553

[28] Cheng E, Blackburn HN, Ng K, Spiegelman D, Irwin ML, Ma X, Gross CP, Tabung FK, Giovannucci EL, Kunz PL, et al. Analysis of Survival Among Adults With Early-Onset Colorectal Cancer in the National Cancer Database. JAMA Netw Open. 2021;4(6):e2112539. 34132794 10.1001/jamanetworkopen.2021.12539 PMC8209612

[29] Barot S, Liljegren A, Nordenvall C, Blom J, Radkiewicz C. Incidence trends and long-term survival in early-onset colorectal cancer: a nationwide Swedish study. Ann Oncol. 2025;36(11):1400–8. 40816336 10.1016/j.annonc.2025.07.019

[30] Jin Z, Dixon JG, Fiskum JM, Parekh HD, Sinicrope FA, Yothers G, Allegra CJ, Wolmark N, Haller D, Schmoll HJ, et al. Clinicopathological and Molecular Characteristics of Early-Onset Stage III Colon Adenocarcinoma: An Analysis of the ACCENT Database. J Natl Cancer Inst. 2021;113(12):1693–704. 34405233 10.1093/jnci/djab123 PMC8634466

[31] Kneuertz PJ, Chang GJ, Hu CY, Rodriguez-Bigas MA, Eng C, Vilar E, Skibber JM, Feig BW, Cormier JN, You YN. Overtreatment of young adults with colon cancer: more intense treatments with unmatched survival gains. JAMA Surg. 2015;150(5):402–9. 25806815 10.1001/jamasurg.2014.3572

[32] Leary JB, Hu J, Leal A, Davis SL, Kim S, Lentz R, Friedrich T, Herter W, Messersmith WA, Lieu CH. Risk Without Reward: Differing Patterns of Chemotherapy Use Do Not Improve Outcomes in Stage II Early-Onset Colon Cancer. JCO Oncol Pract. 2025;21(3):333–40. 39047212 10.1200/OP.24.00159 PMC11925348

[33] Manjelievskaia J, Brown D, McGlynn KA, Anderson W, Shriver CD, Zhu K. Chemotherapy Use and Survival Among Young and Middle-Aged Patients With Colon Cancer. JAMA Surg. 2017;152(5):452–9. 28122072 10.1001/jamasurg.2016.5050 PMC5806125

[34] Zaborowski AM, Murphy B, Creavin B, Rogers AC, Kennelly R, Hanly A, Martin ST, O’Connell PR, Sheahan K, Winter DC. Clinicopathological features and oncological outcomes of patients with young-onset rectal cancer. Br J Surg. 2020;107(5):606–12. 32149397 10.1002/bjs.11526

