# Supplementary material for: Clinicopathological characteristics and stage-adjusted outcomes of sporadic pMMR/MSS early-onset colorectal cancer
Source: BMC Gastroenterol. 2026 May 21;26:445. doi: 10.1186/s12876-026-04938-8 (PMC13366689; doi:10.1186/s12876-026-04938-8)
Supplement: Supplementary file 1 — Supplementary Material 1. [file 12876_2026_4938_MOESM1_ESM.pdf]

Supplementary Table S1. Comparison of baseline clinicopathological characteristics between the included pMMR/MSS study cohort and excluded cases with unknown MMR/MSI status

	**Total**<br><br>**n=1,470**	**pMMR/MSS study cohort**<br><br>**n=1,218 (82.9%)**	**Excluded cases with unknown MMR/MSI status**<br><br>**n=252 (17.1%)**	***P***
Age group				0.587
EOCRC (< 50 years)	101 (6.9%)	86 (7.1%)	15 (6.0%)	
LOCRC (≥ 50 years)	1369 (93.1%)	1132 (92.9%)	237 (94.0%)	
Age				0.128
Mean ± SD	68.2 ± 11.3	68.4 ± 11.3	67.2 ± 10.9	
Sex				0.391
Male	918 (62.4%)	767 (63.0%)	151 (60.0%)	
Female	552 (37.6%)	451 (37.0%)	101 (40.0%)	
Location				**<0.001**
Right colon	432 (29.4%)	374 (30.7%)	58 (23.0%)	
Left colon	573 (40.0%)	483 (39.7%)	90 (35.7%)	
Rectum	465 (31.6%)	361 (29.6%)	104 (41.2%)	
Histological type				0.658
Well–Moderately differentiated	1375 (93.5%)	1137 (93.3%)	238 (94.4%)	
Poorly differentiated	24 (1.6%)	19 (1.6%)	5 (2.0%)	
Mucinous	68 (4.6%)	59 (4.8%)	9 (3.5%)	
Signet-ring cell	3 (0.2%)	3 (0.2%)	0 (0.0%)	
Baseline stage				**<0.001**
Stage 0	61 (4.1%)	49 (4.0%)	12 (4.8%)	
Stage I	381 (25.9%)	277 (22.7%)	104 (41.3%)	
Stage II	358 (24.4%)	319 (26.2%)	39 (15.5%)	
Stage III	477 (32.4%)	398 (32.7%)	79 (31.3%)	
Stage IV	193 (13.1%)	175 (14.4%)	18 (7.1%)	
R0 resection				**0.002**
Yes	1304 (88.7%)	1067 (87.6%)	237 (94.0%)	
No	166 (11.3%)	151 (12.4%)	15 (5.6%)	

pMMR/MSS, mismatch repair-proficient/microsatellite stable; EOCRC, early-onset colorectal cancer; LOCRC, late-onset colorectal cancer; SD, standard deviation.

Supplementary Figure S1

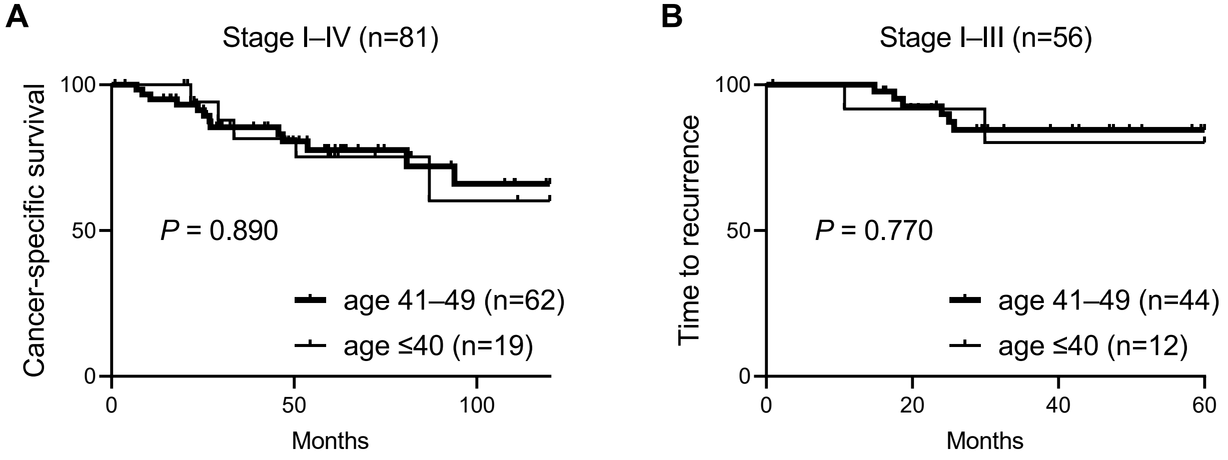

Supplementary Figure S1. Exploratory survival analysis within the EOCRC cohort using a younger age cutoff. Kaplan-Meier curves comparing patients aged ≤40 years and those aged 41–49 years for cancer-specific survival among patients with Stage I–IV disease (A) and time to recurrence among patients with curatively resected Stage I–III disease (B). *P*-values were calculated using the log-rank test.

Supplementary Figure S2

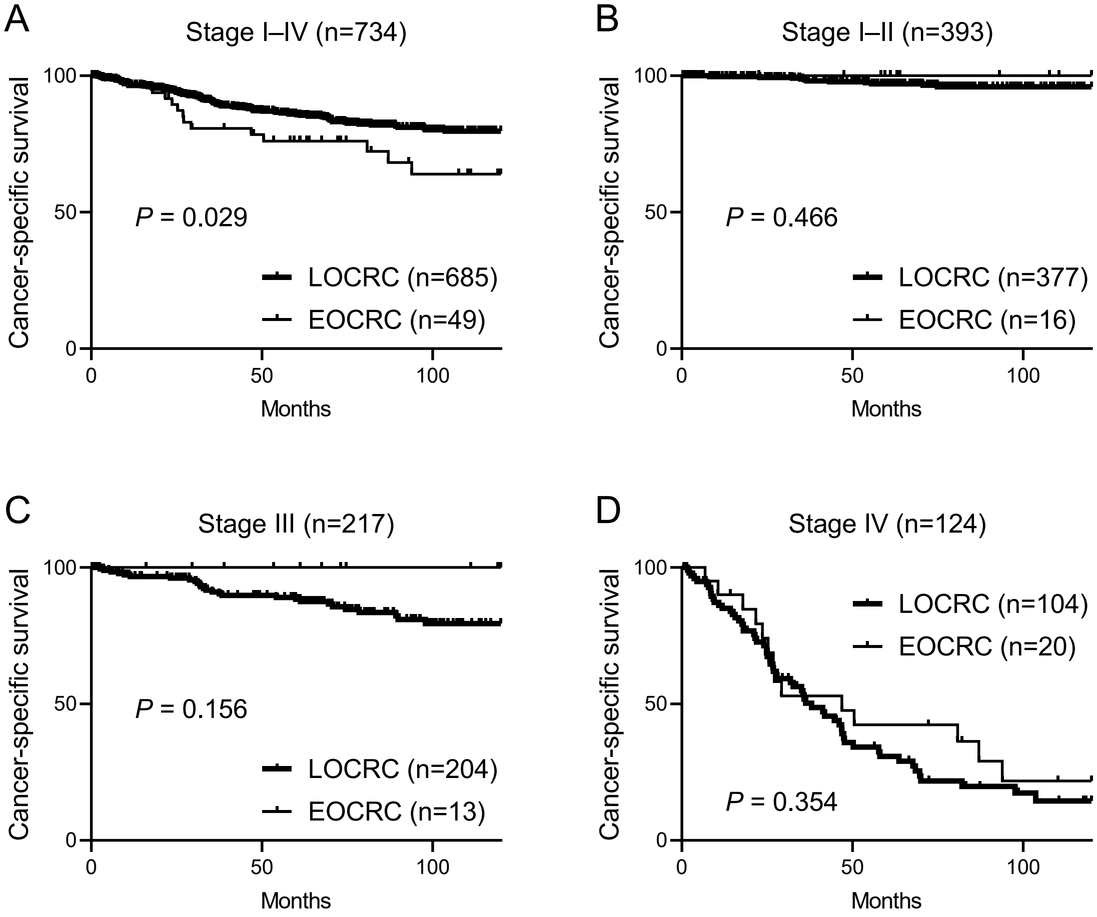

Supplementary Figure S2. Sensitivity analysis of cancer-specific survival in patients with longer follow-up. Kaplan-Meier curves for cancer-specific survival in patients who underwent surgery through 2019, excluding those treated in 2020 or later. Curves compare EOCRC and LOCRC in the overall Stage I–IV cohort (A), and stratified by tumor stage: Stage I–II (B), Stage III (C), and Stage IV (D). *P*-values were calculated using the log-rank test.

Supplementary Figure S3

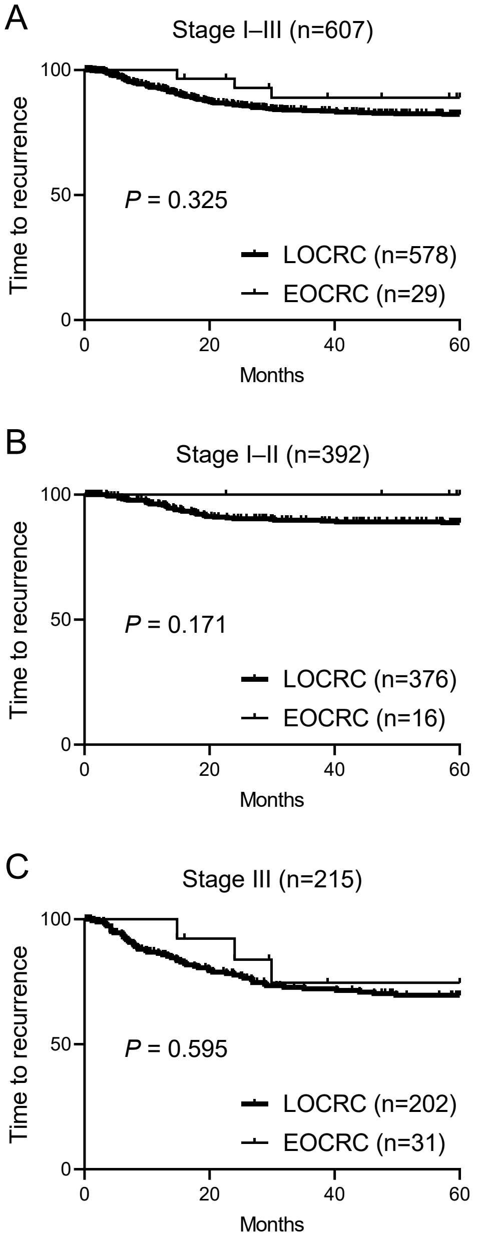

Supplementary Figure S3. Sensitivity analysis of time to recurrence in patients with longer follow-up. Kaplan-Meier curves for time to recurrence among patients with curatively resected Stage I–III disease who underwent surgery through 2019, excluding those treated in 2020 or later. Curves compare EOCRC and LOCRC in the overall Stage I–III disease (A), and subgroup analyses for Stage I–II (B) and Stage III (C). *P*-values were calculated using the log-rank test.
